# Cardiovascular Disease and All-Cause Mortality Among Individuals with Low Sexual Frequency

**DOI:** 10.3390/healthcare13050461

**Published:** 2025-02-21

**Authors:** W. Sumner Davis, Peter Anderson, Sri Banerjee

**Affiliations:** College of Health Sciences, Walden University, Minneapolis, MN 55401, USA; sumner.davis@mail.waldenu.edu (W.S.D.); peter.anderson@mail.waldenu.edu (P.A.)

**Keywords:** sexual health, cardiovascular disease, mortality, sexual dysfunction

## Abstract

**Introduction**: Sexual frequency is an important indicator of overall health and plays a vital role in various health conditions. There is a wide array of physical and mental health benefits that are associated with sexual activity. Cardiovascular disease (CVD) is the most common cause of mortality each year. The purpose of this study was to explore a connection between CVD and all-cause mortality and if sexual frequency modified this effect. **Methods**: For this study, we utilized the 2005–2016 National Health and Nutrition Examination Survey (NHANES) and the NDI-linked all-cause mortality data of US adults aged between 20–59 years. Low sexual frequency was determined by individuals who had sexual intercourse less than once a month. Survival curves showed the combined effect of sexual frequency and all-cause mortality, using the Kaplan–Meier product-limit method to estimate the percent survival of the subject at each point in time. **Results**: For all-cause mortality, the unadjusted hazard ratio (HR) for CVD to no CVD was HR = 5.1. The adjusted HR was elevated HR = 2.3 among individuals who had CVD and low sexual frequency but close to 1.0 among individuals who had a history of CVD but reported moderate-to-high/high sexual frequency after adjusting for demographic and health variables. **Conclusions**: From a nationally representative sample, our study was the first to demonstrate, in unadjusted and adjusted models, that CVD and low sexual frequency combined have worse outcomes than CVD alone. This finding indicates the need to conduct a sexual history among individuals with CVD or other chronic diseases.

## 1. Introduction

Sexual activity, specifically partnered sexual behavior, is an important indicator of overall health and plays a vital role in various health conditions. However, in recent years, physical sexual activity with partners has declined due to the increased choice in sexual outlets, such as social media, pornography consumption, sextech proliferation, and cybersex [1,2,3,4,5]. There is a wide array of physical and mental health benefits that are associated with maintaining regular sexual activity [6]. Given that cardiovascular disease (CVD) remains the leading cause of mortality, the benefits of sexual activity on the risk factors for CVD is especially important to address. The World Health Organization (WHO) estimated that CVD causes approximately 18 million deaths annually [7]. Key risk factors for CVD include hypertension, hyperlipidemia, diabetes, smoking, obesity, and physical inactivity. Researchers have found that there is a close connection between optimal sexual health and the physiological benefits that this offers to the prognosis and outcomes of chronic disease [8]. Optimal sexual health promotes physical and emotional well-being which, in turn, may boost immune function and reduce stress, positively impacting the management of chronic disease. Researchers have found that there is a close connection between sexual health, immune function, and atherosclerosis formation—a major precursor for CVD [9]. Significant studies, including the Caerphilly Cohort Study, US Duke Study, and Corona’s research on Italian men, have explored these connections extensively, highlighting their role in understanding sexual frequency’s impact on cardiovascular outcomes. Significant studies have provided robust insights into the relationship between sexual frequency and cardiovascular health. For instance, the Caerphilly Cohort Study, conducted in Wales, examined long-term cardiovascular outcomes in a cohort of middle-aged men, revealing that higher sexual activity was associated with a reduced risk of coronary heart disease and stroke over a ten-year follow-up period [10]. The US Duke Study built upon these findings by exploring the physiological and psychological benefits of regular sexual activity among both men and women, emphasizing improvements in heart rate variability, stress reduction, and overall cardiovascular fitness [11]. Additionally, Corona’s research on Italian men offered a focused perspective on the metabolic and endocrine factors at play, demonstrating how sexual frequency positively correlates with healthier lipid profiles, lower blood pressure, and improved testosterone regulation [12]. Collectively, these studies underscore the potential protective effects of sexual activity on cardiovascular health, suggesting that frequency may serve as a marker of both physical and emotional well-being.

Declining sexual activity has been correlated with an increased occurrence and effect of chronic diseases. Sexual dysfunction, measured by sexual frequency, is an important indicator of overall health and plays a vital role in various health conditions [10,11]. From a study based on the General Social Survey, researchers found that adults in the early 2010s were having sexual intercourse nine fewer times per year than in the late 1990s, and sexual frequency continues to drop [1,3]. More specifically, married adults were having less sex per year during the same time period. More recently, studies showed an increased proportion (50% in 2009 to 74% in 2018) of adolescents with no sexual activity, including masturbation [12,13,14]. Given the decreasing trends of sexual activity, the precise connection between sexual health and overall wellness is important to ascertain. Recently, studies have emphasized the importance of understanding sex-specific risk factors of CVD and how this is connected to sexual health. In a large cross-sectional study, conducted between April 2022 to August 2023, researchers found that women often experience underdiagnosis and undertreatment in sexual health following CVD, leading to worse long-term cardiovascular outcomes [15,16]. Based on research, an important 10-year prediction scoring system for CVD events (known as Q-RISK3) that has been developed includes erectile dysfunction, migraines, corticosteroid use, atypical antipsychotics, and severe mental illness [17,18,19,20,21]. These conditions were newly updated from previous versions of the Q-RISK prediction tool underscoring the importance of assessing sexual health as a risk factor of cardiovascular disease (CVD) events.

### Fundamental Cause Theory

**Figure 1:**
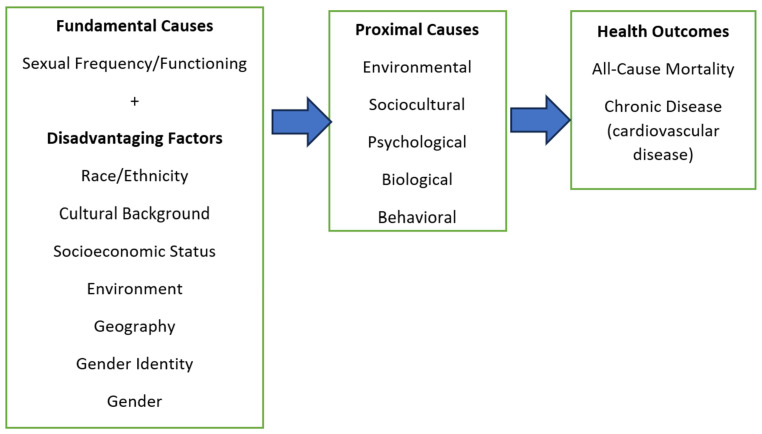
Fundamental Cause Theory and Chronic Disease Outcomes [23].

The Theory of Fundamental Causes [22,23] plays a significant role in this study. As illustrated in Figure 1, this theory seeks to explain why social disparities in health persist despite advancements in medical treatments and the eradication of certain diseases [24]. It encompasses several key concepts emphasizing the influence of social factors on disease development.

The first concept involves contextualizing fundamental individual-based factors to predict variations in disease characteristics among individuals. In this study, we apply this concept to cardiovascular disease (CVD). The second concept focuses on fundamental causes, specifically the social determinants of health, such as socioeconomic status, which influence disease-related risk factors [2022]. In our model, potential moderating variables are categorized as proximal determinants, with health conditions serving as the final outcomes. Additionally, sexual frequency has been incorporated as a moderating variable.

This theory originates from Lieberson’s concept of basic causes, introduced in 1985, which suggests that changes in fundamental causes—factors driving specific outcomes—lead to corresponding changes in those outcomes [22,23,24].

Sexual function is a complex process influenced by hormonal, neurological, psychological, and vascular factors, all of which contribute to overall sexual health. Existing research suggests a link between sexual health and potential therapeutic benefits in managing chronic diseases [8]. However, limited studies have examined the relationship between sexual health, cardiovascular disease (CVD), and long-term mortality outcomes. This study analyzes trends in sexual frequency from 2005 to 2016 and investigates the long-term associations between CVD and all-cause mortality, particularly considering the role of sexual frequency. Additionally, after controlling for physical health factors, such as diabetes, obesity, and hypertension, as well as demographic variables, like age, ethnicity, and education, this study explores whether sexual frequency independently affects all-cause mortality.

## 2. Methods

For this research, we utilized six consecutive two-year cycles (2005–2016) of data from the National Health and Nutrition Examination Survey (NHANES), a program under the National Center for Health Statistics (NCHS) that evaluates the health of non-institutionalized U.S. adults. The study sample is representative of adults aged 20 to 59 years and follows the Strengthening the Reporting of Observational Studies in Epidemiology (STROBE) guidelines. Before the data collection, the NHANES procedures were approved by the NCHS. The publicly available data were obtained through the Centers for Disease Control and Prevention (CDC) website. Additionally, ethical approval for the data analysis was granted by the Walden University Institutional Review Board.

### 2.1. All-Cause Mortality

Mortality status was determined using the Continuous NHANES Public-Use National Death Index (2005–2016), which tracks participants’ survival status in person-months from their NHANES survey participation date until death or 31 December 2019. This linked mortality file utilized probabilistic matching to connect NHANES participants with death records. 

### 2.2. CDC Mortality Linkage

#### 2.2.1. Measures: Cardiovascular Disease (CVD)

CVD was identified using the Medical Conditions Questionnaire (MCQ), which includes self-reported diagnoses of the following:

1. Congestive heart failure—“Has a doctor or other health professional ever diagnosed you with congestive heart failure?”
2. Coronary heart disease—“Has a doctor or other health professional ever diagnosed you with coronary heart disease?”
3. Angina (angina pectoris)—“Has a doctor or other health professional ever diagnosed you with angina?”
4. Heart attack (myocardial infarction)—“Has a doctor or other health professional ever diagnosed you with a heart attack?”
5. Stroke—“Has a doctor or other health professional ever diagnosed you with a stroke?”

Participants who answered “yes” to any of these questions were classified as having CVD.

#### 2.2.2. Measures: Sexual Frequency and Sexual Dysfunction

Sexual dysfunction was assessed based on sexual frequency, which served as the primary indicator. This study primarily examined the frequency of sexual interactions, as self-reported during NHANES in-person interviews. Participants were asked the following:

“In the past 12 months, how many times have you had vaginal or anal sex?”

Response categories included “Never”, “Once”, “2–11 times”, “12–51 times”, “52–103 times”, “104–364 times”, or 365 times or more.

For analyzing the association between sexual activity and mortality outcomes, the participants were grouped into two categories: less than 11 times per year (<11 times/year) and 11 or more times per year (≥11 times/year), a previously validated threshold [21]. The dataset also allowed for the further stratification of sexual frequency, such as 12–30 times/year and 31–50 times/year, providing more detailed insights beyond the basic binary classification.

#### 2.2.3. Measures: Obesity

Obesity classification was based on the body mass index (BMI), calculated using measured height and weight. Participants were categorized as follows: “BMI < 25: Moderate to high weight”; “BMI 25–29: Overweight”; or BMI ≥ 30: Obese or severely obese. For the statistical analysis, obesity was considered present (BMI ≥ 30) or absent (BMI < 30) in both univariate and multivariate models.

#### 2.2.4. Measures: Diabetes

Participants aged 20 or older were asked the following:

“Other than during pregnancy, has a doctor or other health professional ever told you that you have diabetes or sugar diabetes?”

Those who responded “yes” or “borderline” were classified as having diabetes, while those who answered “no” were considered not to have diabetes.

#### 2.2.5. Measures: Smoking Status

Self-reported smoking status was determined based on lifetime cigarette consumption and current smoking behavior. Participants who reported smoking at least 100 cigarettes in their lifetime were classified as smokers, while those who had smoked fewer than 100 cigarettes were categorized as never smokers.

Current smoking status was further assessed with the question:

“Do you currently smoke cigarettes?” Participants who answered “every day” or “some days” were categorized as current smokers. Those who answered “not at all” were classified as former or non-smokers.

#### 2.2.6. Measures: Additional Covariates

Hypertension was identified based on self-reported data. Participants who reported having hypertension were classified as hypertensive, while the rest were considered non-hypertensive. Demographic variables such as age and ethnicity were included as covariates. Ethnicity was categorized as “Non-Hispanic White”, “Non-Hispanic Black”, “Hispanic”, or “Other”. Education level was classified into three groups, “ Some high school”, “High school graduate”, or “Some college or higher”. Marital status was categorized as ”Married”, “Widowed”, “Divorced”, “Separated”, “Never Married”, and “living with partner”. Income level was determined using the family monthly poverty index, where a value of <1.00 indicated low income.

## 3. Results

**Figure 2:**
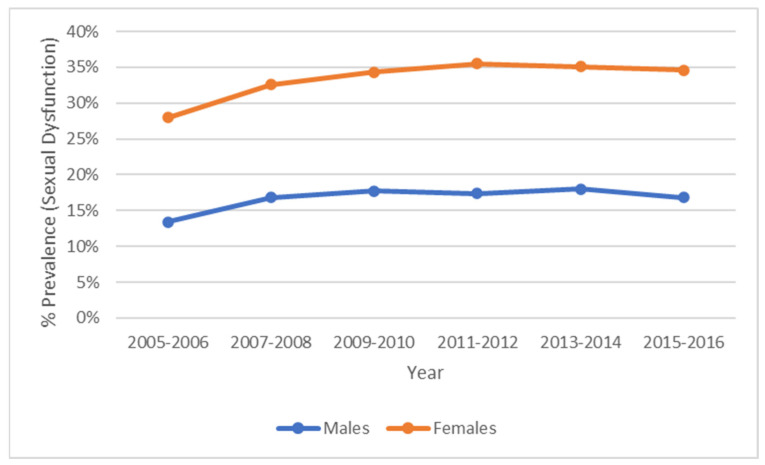
Low sexual frequency (sexual dysfunction) prevalence from 2005–2016.

**Figure 3:**
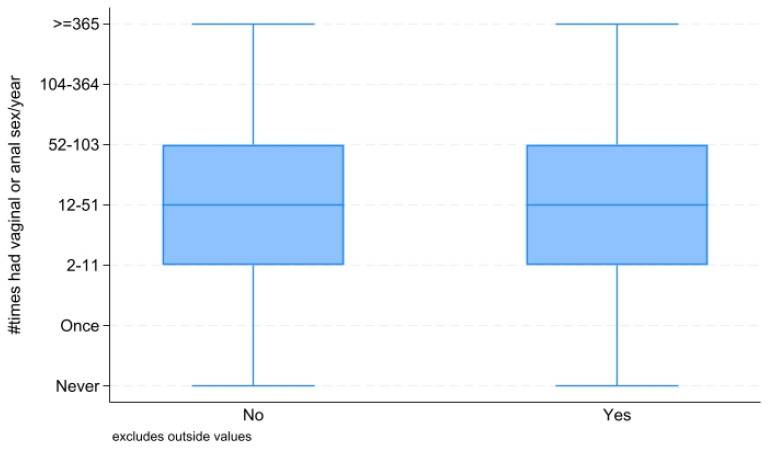
Graded sexual frequency stratified by CVD status.

After combining data from the NHANES 2005–2016, we had an unweighted sample size of 15,812 individuals (weighted n = 193.7 million) aged 20 to 59 years with complete medical examination data. As shown in Figure 2, between 2005–2016, low sexual frequency increased for both males (13.4% to 16.8%) and females (14.6% to 17.8%). As seen in Table 1, less than half (46%) had a low sexual frequency and more than 3% had CVD. Table 1 displays the demographic characteristics of the sample. Approximately 49.7% were women, 66.5% were non-Hispanic white, and their mean age was 42.0 years. In the trend analysis, low sexual frequency has increased for both males (13.4% to 16.8%) and females (14.6% to 17.8%). According to Figure 3, the mean frequency was demonstrated to be the same for people with CVD versus without CVD. Also, when dichotomized, people with a low sexual frequency are likely to have CVD, be obese, hypertensive, diabetic, and to smoke. As shown in Table 1, upon mean mortality follow-up, people with a low sexual frequency experienced an increased (4.0% vs. 2.0%, *p* < 0.001) proportion of mortality compared to individuals with a moderate-to-high sexual frequency. Also, among those with a low sexual frequency, a higher (4.9% vs. 3.5%, *p* < 0.01) proportion of males died than females.

**Figure 4:**
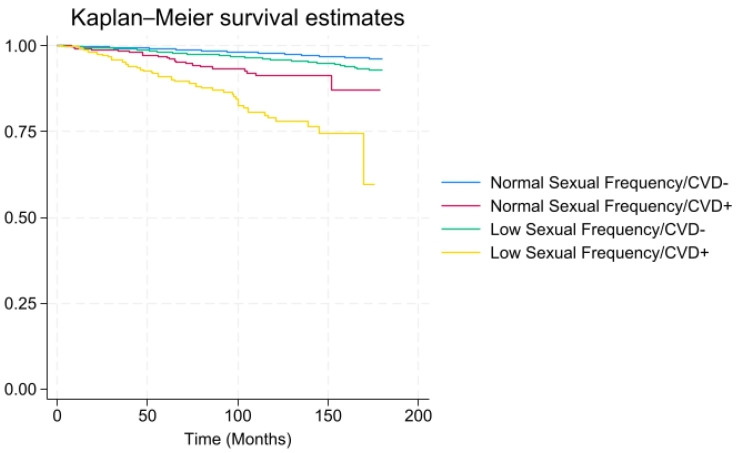
All-cause mortality among individuals stratified by sexual frequency (low vs. moderate-to-high/high sexual frequency)/CVD (*x*-axis: time in months; *y*-axis: overall survival probability).

For all-cause mortality, the unadjusted hazard ratio (HR) for CVD to no CVD was 5.05 (95% confidence interval [CI], 3.46–7.38, *p* < 0.001). Even after adjustment, the HR for CVD to no CVD remained strong (HR = 1.73) As seen in Table 2, the adjusted HR was elevated 2.26 (CI 1.20–4.26, *p* < 0.05) among individuals who had CVD and a low sexual frequency, but close to 1.0 (1.21 CI 0.60–2.43, *p* = 0.59) among individuals who had a history of CVD but reported a moderate-to-high sexual frequency after adjusting for demographic (ethnicity, education, poverty-income-ratio, age, marital status, and gender) and health variables (hypertension, obesity, diabetes, and smoking status). As shown in Figure 4, there was a higher proportion of all-cause mortality over time among individuals with both a low sexual frequency and CVD than each variable independently. For instance, at 100 months (8.3 years), there was 30% mortality from individuals that had both a low sexual frequency and CVD, rather than each variable (low sexual frequency-5% or CVD-15% mortality) individually.

As seen in Table 2, upon stratified analysis, among all the variables, sexual frequency modified the relationship between gender, diabetes, and poverty and all-cause mortality. First of all, compared to those without diabetes, the adjusted hazard ratio (HR) for the all-cause mortality risk among those with diabetes was 1.69 (95%CI = 1.21–2.37, *p* < 0.01). The adjusted HR was elevated, 1.77 (CI 1.17–2.68, *p* < 0.01), among individuals who had a history of diabetes and low sexual frequency, but close to 1.0 (1.52 CI 0.91–2.54) among individuals who had diabetes but reported a moderate-to-high sexual frequency, after adjusting for medical and demographic risk factors. Comorbidities such as diabetes play a vital role in determining all-cause mortality.

Also, in comparison to females, the adjusted hazard ratio (HR) for the all-cause mortality risk among males was 1.41 (95%CI = 1.11–1.80, *p* < 0.01). The adjusted HR was elevated, 1.57 (CI 1.08–2.28, *p* < 0.05), among males with a low sexual frequency, but close to 1.0 (1.35 CI 0.96–1.89) among males reporting a moderate-to-high sexual frequency, after adjustment. Finally, compared to those with a moderate-to-high income, the adjusted hazard ratio (HR) for the all-cause mortality risk among those with a low income was 1.58 (95%CI = 1.13–2.21, *p* < 0.01). The adjusted HR was elevated, 1.94 (CI 1.15–3.26, *p* < 0.05), among impoverished individuals with a low sexual frequency, but close to 1.0 (1.33 CI 0.86–2.05) among impoverished individuals who reported a moderate-to-high sexual frequency, after adjusting for medical and demographic variables.

## 4. Discussion

In this nationally representative cohort, we found that between 2005–2016, sexual dysfunction increased 25% for males and 22% for females. This increase trend has been reflected in research conducted in various populations previously. We also found that females have a higher percentage of sexual dysfunction than males. According to a previous cross-sectional study, researchers found that females experience more (43% vs. 31%) sexual issues than males [25]. Additionally, while Li et al. [26] found that mental dysfunction has an impact on sexual frequency, Davis et al. [27] found that the common denominator between the connection of chronic diseases and poor sexual health is chronic inflammation, which may be counteracted through higher sexual frequency. Finally, researchers found that there is a strong connection between CVD and sexual behavior due to increased inflammation that simultaneously affects both [28,29].

Another novel finding was that when individuals experience low sexual frequency, they have a higher proportion of all-cause mortality than the general population. We also found that sexual frequency is directly associated with CVD prevalence, making the underlying mechanisms of utmost importance. According to previous researchers, medications can have significant effects on libido (sexual desire) and sexual frequency, primarily by influencing hormonal balance, neurological function, or physical processes necessary for sexual activity [30,31,32,33,34]. The mechanisms of interference include hormonal disruption through impacts on levels of testosterone, estrogen, and other hormones critical to both sexual function and CVD, often resulting in a decrease in libido or sexual desire [31,34]. While these findings are significant, it is important to acknowledge existing literature that has previously explored similar connections, such as the studies by Corona et al., the Caerphilly Cohort, and other longitudinal analyses [10,11,12].

We also found that after controlling for confounders, low sexual frequency alone is correlated with an increased likelihood of mortality. Additionally, when the CVD and low sexual frequency status was positive, there was a higher overall mortality than those individuals that have low sexual frequency or CVD individually. To our knowledge, this was the first time that anyone has found, through mortality follow-up, that there is a connection between chronic disease and sexual health. The cardiovascular system plays a central role in sexual health by ensuring adequate blood flow to the genital organs, especially in male penile engorgement [35]. Inflammation, allostatic load, and hormonal imbalance can all be mechanisms through which chronic diseases are connected sexual dysfunction.

In addition, we found that sexual frequency modifies the effect of gender, diabetes, and poverty on all-cause mortality. Individuals that were poor and had a low sexual frequency had a higher probability to die than those individuals that were poor but had a moderate-to-high sexual frequency. Also, individuals with diabetes and a low sexual frequency were more likely to die than individuals with either condition alone. Obesity and diabetes are a constellation of comorbidities that are a part of metabolic syndrome, a major risk factor for CVD. Another study on sexual dysfunction revealed that obesity, interrelated with diabetes, had higher rates of coital pain, sexual dissatisfaction, and arousal problems [19]. Also, we found that compared to females, males with a low sexual frequency and CVD had a higher probability of mortality than those males with each individual risk factor. These connections with social variables and chronic diseases underscore the importance of assessing for corollary conditions along with sexual frequency [18,19,24]. Socioeconomic disparities have repeatedly been shown to impact allostatic load, that can, in turn, be connected to low sexual frequency and dysfunction.

Due to our findings being related to sexual behavior and chronic diseases, there is a need to explore underlying mechanisms that relate the two conditions. In men, erectile dysfunction (ED) is one of the most common manifestations of sexual dysfunction, affecting up to 50% of men over the age of 50 [35]. ED in men is often associated with endothelial dysfunction, which reduces nitric oxide availability and impairs vasodilation, ultimately limiting blood flow to the penis and, simultaneously, is an early indicator of CVD [36]. The process of erection in men is heavily dependent on blood flow to the penis, which is mediated by the dilation of blood vessels and the relaxation of smooth muscle [37,38,39]. In men, erectile dysfunction (ED) often serves as an early indicator of underlying cardiovascular issues, including endothelial dysfunction, which could signal a higher risk of heart disease [40,41,42]. In another research study, Wu et al. [21] concluded that the severity of ED correlates with various aspects of CVD, including the number of coronary stenoses and the severity of atherosclerosis. The researchers confirmed that addressing ED can also aid in managing cardiovascular risk, given its association with underlying conditions like hypertension, diabetes, and mental health factors such as anxiety and depression [21]. This relationship highlights the bidirectional nature of sexual dysfunction and CVD, suggesting that interventions targeting ED could play a preventive role in CVD progression.

Sexual dysfunction related to CVD, among women, is often characterized by decreased libido, arousal difficulties, and orgasmic dysfunction. Sexual arousal involves increased blood flow to the genital region, which facilitates lubrication and enhances sexual pleasure [39]. Research indicates that women with heart disease experience a significant decline in sexual desire and satisfaction compared to healthy individuals [40]. Factors such as reduced blood flow, hormonal imbalances (e.g., menopause-related estrogen decline), and psychological stress all contribute to sexual dysfunction in women with cardiovascular conditions [39]. Any impairment in the cardiovascular system, such as that seen in CVD, can disrupt these processes and lead to sexual dysfunction [41]). Given this relationship, sexual dysfunction can be an early and sensitive indicator of cardiovascular health [42].

### 4.1. Recommendations

There are major practical implications about applying sexual behavior and assessing the connection with CVD. Researchers have found that optimal sexual health can lead to physiological benefits in improving prognosis and outcomes of chronic disease. Optimal sexual health promotes physical and emotional well-being which, in turn, boosts immune function and reduces stress, positively impacting the management of chronic disease [20]. However, there is a need to have consistency in the way that sexual health is viewed by different organizations—on a personal level and a population level. Screening for sexual dysfunction should be administered for those individuals that are diagnosed with chronic diseases. In addition, health care professionals should create an environment and treat sexual dysfunction before this starts to severely impact overall health. Also, it is of paramount importance for health care practitioners to have dialogue about the sexual side effects of medications [35]. By gauging the comfort level of the patient, healthcare practitioners can assess the readiness to converse about these issues and reduce stigma.

There is a critical need for consistency in how sexual dysfunction is defined and added as recommendations and goals for the nation. Major public health organizations focus on promoting safe, consensual, and satisfying sexual experiences, but they do not specifically address the frequency of sexual activity in relation to mortality or cardiovascular health. When comparing the definitions of sexual health, there are discrepancies in how sexual health is viewed. Both the Centers for Disease Control and Prevention (CDC) and the World Health Organization (WHO) have not reported on the direct correlation between low sexual frequency and increased mortality, either independently or in conjunction with cardiovascular disease (CVD) [10,11]. In comparison, the WHO defines sexual health as a state of physical, emotional, mental, and social well-being related to sexuality. The standardization of the definitions can lead to increased screening and sexual satisfaction on a population level, resulting in improved outcomes.

### 4.2. Limitations

The results suggest that patients with CVD, who experience low sexual frequency, have a higher probability of death than those individuals with CVD or low sexual frequency alone. However, it was difficult to ascertain if low sexual frequency came before or after the diagnosis of CVD or vice versa, because the time of diagnosis was not present. The assumption can be made that poor sexual frequency was experienced more recently than CVD was based on what is experienced in the previous year. In addition, due to these being self-reported data, this study was prone to recall bias, due to the use of self-reported data, and social desirability bias. Also, there are alternative ways to calculate obesity, like waist circumference rather than BMI, that may render more accurate results. In addition, there was no way to objectively determine if the patient had CVD, but this has been shown to be an equivalent correlation to physician diagnoses.

## 5. Conclusions

CVD has a profound impact on sexual health, affecting both men and women. Sexual dysfunction, including erectile dysfunction and reduced sexual satisfaction, often serves as an early indicator of cardiovascular problems. The findings support a multidisciplinary approach to CVD care that includes cardiologists, sexologists, and mental health professionals to ensure comprehensive patient support. Understanding the complex relationship between cardiovascular health and sexual function is critical for providing comprehensive care to patients with CVD. The improved integration of sexual health in cardiovascular care could include routine assessments of sexual function, targeted therapies for sexual dysfunction, and patient education on safe sexual activity post-CVD events. Current treatments, including pharmacological interventions, lifestyle modifications, and psychological support, can significantly improve sexual function. As research progresses, new therapies and interventions hold the potential to enhance sexual health outcomes for individuals with cardiovascular disease.

## Figures and Tables

**Table 1:** Characteristics of Study Participants aged 20 to 59 with CVD and Low Sexual Frequency.

Characteristics	Total Population (n = 10,596)	Low Sexual Frequency (Less Than Once a Month) (n = 4840)	Moderate-to-High/High Sexual Frequency (Once a Month or More) (n = 5756)
CVD (%) **	3.1 (2.8–3.5)	4.9 (4.3–5.6)	2.4 (2.1–2.8)
Smoking Status (%) **			
Never Smoked	64.9 (63.5–36.6)	63.0 (61.3–64.8)	65.7 (64.1–67.3)
Formerly Smoked	19.4 (18.5–20.2)	19.9 (18.6–21.2)	19.1 (18.1–20.2)
Currently Smoke	15.7 (14.7–16.7)	17.1 (15.7–18.5)	15.1 (14.1–16.2)
Hypertension (%) **	21.5 (20.4–22.6)	25.4 (23.7–27.20	19.9 (18.8–21.1)
Diabetes (%) **	6.4 (5.9–7.0)	9.3 (8.2–10.4)	5.3 (4.8–5.9)
Age (SE) **	38.5 (0.20)	41.7 (0.27)	36.1 (0.22)
Gender Male (%)	50.3 (49.5–51.1)	51.5 (49.9–53.1)	49.8 (48.9–50.8)
Marital Status (%) **			
Married	58.7 (56.9–60.5)	50.0 (47.052.3)	62.2 (60.5–64.0)
Widowed	0.7 (0.5–0.8)	1.4 (1.0–1.9)	0.4 (0.3–0.5)
Divorced	8.1 (7.5–8.7)	11.6 (10.3–12.9)	6.7 (6.1–7.3)
Separated	2.4 (2.1–2.8)	3.3 (2.8–3.9)	2.1 (1.7–2.5)
Never Married	19.1 (17.5–20.6)	25.9 (23.7–28.2)	16.4 (14.9–17.8)
Living with Partner	11.1 (10.3–11.9)	8.2 (7.2–9.2)	12.3 (11.3–13.2)
Ethnicity (%) **			
Non-Hispanic White	66.5 (63.6–69.4)	62.4 (59.0–65.7)	68.1 (65.2–71.0)
Non-Hispanic Black	11.5 (10.0–13.0)	15.0 (13.0–17.0)	10.1 (8.7–11.5)
Hispanic	15.2 (13.1–17.3)	16.1 (13/9–18.3)	14.8 (12.6–17.0)
Other	6.9 (6.2–7.6)	6.5 (5.5–7.7)	7.0 (6.3–7.8)
Education Level (%) **			
Some High School	15.1 (13.6–16.5)	18.4 (16.3–20.6)	13.7 (12.4–15.1)
High School Graduate	21.3 (20.0–22.6)	22.7 (20.9–24.5)	20.8 (19.3–22.2)
Some College and Beyond	63.6 (61.3–65.9)	58.9 (55.8–61.9)	65.5 (63.2–67.7)
PIR < 1 **	14.2 (12.9–15.4)	17.0 (15.3–18.7)	13.1 (11.8–14.2)
All deaths (N, %) **	(2.6%) 448	(4.0%) 210	(2.0%) 238

Note. ** *p* < 0.01. Numbers with 95CI indicate 95% confidence intervals for proportions.

**Table 2:** Risk of all-cause mortality among individuals aged 20–59 with CVD/low sexual frequency.

	Total Population HR (95%CI)	CVD- Low Sexual Frequency HR (95%CI)	CVD+ Moderate-to-High/High Sexual Frequency HR (95%CI)	CVD+ Low Sexual Frequency HR (95%CI)
CVD/Sexual Frequency	1.73 (1.08–2.76) *	1.06 (0.81–1.40)	1.21 (0.60–2.43)	2.26 (1.20–4.26) *
Smoking Status				
Never Smoked	Ref	Ref	Ref	Ref
Formerly Smoked	1.21 (0.82–1.77)	1.11 (0.71–1.72)	1.13 (0.72–1.77)	1.42 (0.79–2.56)
Currently Smoke	2.88 (2.14–3.87) **	3.06 (2.19–4.27) **	3.23 (2.16–4.83) **	2.47 (1.56–3.92) **
Obesity	1.10 (0.85–1.42)	1.17 (0.89–1.54)	1.07(0.76–1.51)	1.16(0.71–1.89)
Hypertension	1.62 (1.20–2.20) **	1.65 (1.19–2.28) **	1.73(1.12–2.67) *	1.44(0.93–2.24)
Diabetes	1.69 (1.21–2.37) **	1.69 (1.16–2.45) **	1.52(0.91–2.54)	1.77(1.17–2.68) **
Age	1.06 (1.05–1.08) **	1.06 (1.04–1.08) **	1.06 (1.04–1.08) **	1.07 (1.04–1.09) **
Gender (ref. female)	1.41 (1.11–1.80) **	1.40 (1.08–1.81) *	1.35 (0.96–1.89)	1.57 (1.08–2.28) *
Marital Status				
Married	Ref	Ref	Ref	Ref
Widowed	0.94 (0.43–2.03)	0.70 (0.27–1.85)	0.39 (0.05–2.75)	1.11 (0.50–2.46)
Divorced	1.55 (1.04–2.31) *	1.52 (1.01–2.29) *	1.34 (0.71–2.53)	1.70 (0.96–2.99)
Separated	2.24 (1.16–4.30) *	2.15 (1.05–4.43) *	2.44 (1.10–5.41) *	1.95 (0.74–5.15)
Never Married	2.08 (1.40–3.10) **	2.12 (1.40–3.22) **	2.14 (1.29–3.56) **	1.90 (1.11–3.27) *
Living with partner	1.79 (1.16–2.76) **	1.77 (1.11–2.82) *	1.82 (1.04–3.21) *	1.69 (0.91–3.13)
Ethnicity				
Non-Hispanic White	Ref	Ref	Ref	Ref
Non-Hispanic Black	1.07 (0.84–1.36)	1.08 (0.84–1.38)	1.46 (0.99–2.15)	0.72 (0.49–1.05)
Hispanic	0.64 (0.47–0.87) **	0.61 (0.43–0.85) **	0.74 (0.47–1.15)	0.53 (0.35–0.80) **
Other	0.48 (0.23–0.99) *	0.51 (0.24–1.12)	0.44 (0.15 = 1.30)	0.51 (0.19–1.39)
Education Level				
Some College or Beyond	Ref	Ref	Ref	Ref
Some High School	0.67 (0.47–0.96) *	0.61 (0.40–0.92) *	0.66 (0.39–1.11)	0.72 (0.45–1.15)
High School Graduate	0.67 (0.50–0.90) **	0.60 (0.43–0.82) **	0.67 (0.43–1.0–3)	0.71 (0.47–1.06)
PIR < 1	1.58 (1.13–2.21) **	1.52 (1.04–2.22) *	1.33 (0.86–2.05)	1.94 (1.15–3.26) *

Note. * *p* < 0.05, ** *p* < 0.01. HR (95CI) indicates hazard ratios with 95% confidence intervals for the outcome (i.e., mortality). Ref indicates the reference group among each variable for comparison with other groups.

## Data Availability

Data available from the CDC website by National Center for Health Statistics.
